# Multimorbidity patterns and associated factors in older Chinese: results from the China health and retirement longitudinal study

**DOI:** 10.1186/s12877-022-03154-9

**Published:** 2022-06-01

**Authors:** Quan Zhang, Xiao Han, Xinyi Zhao, Yue Wang

**Affiliations:** 1 National School of Development, Peking University, No.5 Yiheyuan Road, Beijing, 100872 China; 2 School of Health Humanities, Peking University, No. 38 Xueyuan Road, Beijing, 100191 China

**Keywords:** Multimorbidity pattern, Associated factors, Elderly population

## Abstract

**Background:**

This study aimed to investigate multimorbidity patterns and their associated factors among elderly population in China.

**Methods:**

A total of 10,479 participants aged at least 60 years were drawn from the 2018 wave of the China Health and Retirement Longitudinal Study (CHARLS). Latent class analysis (LCA) was performed to identify distinct multimorbidity classes based on 14 self-reported chronic conditions. The multinomial logit model was used to analyze the associated factors of multimorbidity patterns, focusing on individuals' demographic characteristics, socioeconomic status (SES), and health behaviors.

**Results:**

Among the 10,479 participants (mean age [SD]: 69.1 [7.1]), 65.6% were identified with multimorbidity. Five multimorbidity clusters were identified by LCA: relatively healthy class (49.8%), vascular class (24.7%), respiratory class (5.6%), stomach-arthritis class (14.5%), and multisystem morbidity class (5.4%). Multinomial logit analysis with the relatively healthy class as the reference showed that participants of older age and female sex were more likely to be in the vascular class and multisystem morbidity class. The probability of being in the vascular class was significantly higher for those with high SES. Ever smoking was associated with a higher probability of being in the respiratory class and multisystem morbidity class. Physical activity was associated with lower odds of being assigned to the vascular class, respiratory class, and multisystem class.

**Conclusion:**

The distinct multimorbidity patterns imply that the prevention and care strategy should target a group of diseases instead of a single condition. Prevention interventions should be paid attention to for individuals with risk factors.

**Supplementary Information:**

The online version contains supplementary material available at 10.1186/s12877-022-03154-9.

## Introduction

Multimorbidity, the co-existence of two or more chronic conditions [1], has become a global public health concern. Multimorbidity can cause adverse health outcomes, including frailty [2], disability [3], and even mortality [4]. It also increases healthcare use and costs [5], challenging healthcare systems around the world [6].

Given its importance, there has been a significant increase in studies on multimorbidity in both developed and developing countries, investigating the prevalence, risk factors, and/or outcomes. However, the measurement of multimorbidity varied in different studies. A large number of prior studies assessed multimorbidity by either the co-existence of two or more conditions or simply counting the number of concurrent conditions [7]. As the co-occurrence of chronic conditions in a single individual is often due to their shared etiology [8, 9], understanding the clusters of multimorbidity may improve the effectiveness of the traditional clinical practice, which treats each condition individually [10, 11].

Some scholars have tried to use cluster analysis approaches, such as latent class analysis (LCA), to investigate multimorbidity patterns. LCA is an appropriate tool to identify latent classes of observed variables based on structural equation modeling. It has been applied by some studies to examine how individuals are clustered according to patterns of chronic conditions. For example, by focusing on 10 self-reported conditions from the National Health and Aging Trends Study in the United States, Nguyen and colleagues [2] identified five multimorbidity classes, including minimal, cardiovascular, osteoarticular, neuropsychiatric, and multisystem. Another study on the middle-aged and older adults in Spain identified three patterns: healthy, metabolic/stroke, and cardiorespiratory/mental/arthritis [12].

However, the clustering patterns of chronic conditions were rarely examined in China using national-level data. Among the few exceptions, some studies used China Health and Retirement Longitudinal Study (CHARLS), a nationally representative dataset in China. A study on individuals aged 50 and older in China identified 76 dyadic and 169 triadic combinations using observed-to-expected ratios [13], which are likely due to a single disease's high prevalence, and have limited practical implications due to the large number of dyads and triads. Another study revealed four multimorbidity patterns among Chinese people aged 45 and older [14]. It focused on the similarities between diseases using hierarchical cluster analysis instead of reporting the rate of different multimorbidity categories. Xu et al. used cluster analysis to identify multimorbidity patterns among the middle-aged population and used early data in 2011 [15]. Moreover, although existing studies on elderly population in China have also shown that multimorbidity was associated with older age, females, low education attainment, and unhealthy behaviors like smoking and alcohol consumption [16, 17], the risk factors for specific multimorbidity patterns have been rarely examined.

The multimorbidity concern could be a bigger problem for developing countries, as their healthcare systems may not be well prepared for the rapid transition from infectious diseases to non-communicable diseases [18]. In addition, China constitutes the largest elderly population globally, with more than 264 million (about 18.7% of the population) aged 60 and older, according to the latest national census in 2020 [19]. The rapid aging process and its accompanied high prevalence of multimorbidity in China may exacerbate the burden of multimorbidity. There are three main public medical insurance schemes in China, namely, the urban employee basic medical insurance (UEBMI) for urban employees and retirees, the urban resident basic medical insurance (URBMI) for the unemployed residents in urban areas, and the new rural cooperative medical scheme (NRCMS) for the rural population [20]. Despite the substantial increase in the coverage of social medical insurance, benefits vary dramatically across these schemes [21]. UEBMI has a higher reimbursement rate and service coverage than URBMI or NRCMS. Meanwhile, the health reform in China has not paid particular attention to the potential burden of multimorbidity [22]. Unlike developed countries which have proposed guidelines for the management of multimorbidity [23], there have been no such guidelines [24]. Therefore, research on multimorbidity patterns and their risk factors among elderly population in China is necessary, which can provide implications for clinical practice and health policy. The present study aims to identify predominant multimorbidity patterns using LCA among community-dwelling elderly population in China and analyze associated factors for certain multimorbidity patterns.

## Methods

### Data and sample

Data were from CHARLS, a nationally representative longitudinal survey in China. CHARLS, harmonized with the Health and Retirement Study (HRS) international family of surveys, is a nationally representative survey of people aged 45 years old or above in China. The strict adherence to stratified probability proportional to size (PPS) random sampling principles and strict quality control measures lead to its representativeness of China (individuals from nursing homes or hospital care facilities also included), as shown by the close match between the 2011 baseline wave and the 2010 census in demographics. CHALRS collects high-quality multidisciplinary data, including basic demographics, socioeconomic status (SES), and health information [25]. The baseline survey in 2011 included 17,708 respondents [25], and all respondents were followed in 2013, 2015, and 2018. By 2013, there were 15,184 follow-up respondents and 3,426 new respondents; by 2015, there were 17,276 follow-up observations and 3,824 new observations; and by 2018, there were 19,188 follow-up observations and 628 new observations.

This study used the 2018 wave of CHARLS in order to provide a more recent picture of multimorbidity patterns in China. We focused on participants aged 60 and above (*n* = 10,794) as the age of 60 is widely considered the onset of old age in China. To study multimorbidity patterns, we deleted those with missing information for any of the 14 chronic diseases (45 dropped), leading to a total of 10,749 participants for descriptive analysis and the identification of multimorbidity patterns (See Supplementary Fig. 1 for a detailed sample selection process).

### Identification of chronic conditions and multimorbidity

In CHARLS, the presence of chronic disease was assessed by the question, "Have you been diagnosed by a doctor with the following conditions?" There were 14 chronic conditions in total, including hypertension, dyslipidemia, diabetes, cancer, chronic lung diseases (such as chronic bronchitis, emphysema), chronic liver disease (except for fatty liver, benign tumors, and cancer), heart problems (i.e., heart attack, coronary heart disease, angina, congestive heart failure), stroke, kidney disease (except for tumor or cancer), stomach or other digestive diseases (except for benign tumor or cancer), emotional, nervous or psychiatric problems, memory-related disease, arthritis or rheumatism, and asthma. To provide a more comprehensive analysis of multimorbidity and be consistent with the literature, we included all 14 conditions in the assessment of multimorbidity [13, 14]. Each condition was coded as a dichotomous variable, with the presence of disease = 1. Multimorbidity was defined as the presence of at least two of the 14 chronic diseases in a single individual. In addition, we used all 14 conditions to identify multimorbidity patterns by latent class analysis (LCA) (see Statistical Analysis section).

### Potentially associated factors

Guided by the literature [17, 26], independent variables involved a list of sociodemographic characteristics and lifestyle factors. For demographics, we included age (in years), sex (female vs. male), and marital status (married vs. unmarried). SES included current residence (urban vs. rural), education (below primary vs. primary and above), household per capita income quintile, and types of medical insurance. There are three main social medical schemes in China – UEBMI, URBMI, and NRCMS [20]. In 2016, the government decided to integrate URBMI and NRCMS into the new Urban–Rural Resident Medical Insurance (URRMI) [27]. However, the integration had not been completed throughout the country by 2018 (i.e., the time of CHARLS survey). Therefore, we classified the participants covered by the URBMI and URRMI into the resident medical insurance group. Hence, the types of medical insurance in this analysis included no medical insurance (MI), UEBMI, resident MI, NRCMS, and other MI (such as private MI). Three lifestyle factors were included: smoking, alcohol consumption, and physical activities. Smoking was defined as a dummy variable, which equals 1 if an individual was a current or past smoker, and 0 if an individual has never smoked. Alcohol consumption was also defined as a dummy variable (never vs. past or current). Physical activity was a categorical variable, measuring the highest intensity of weekly physical activity (no physical activities, mild activities such as walking, moderate activities such as bicycling at a normal speed, Tai-Chi, etc., and vigorous-intensity activities such as carrying heavy stuff, aerobic workout, bicycling at a fast speed, etc.).

When investigating the associated factors of multimorbidity patterns, we imputed all categorical independent variables by adding a missing category for each independent variable to retain the maximum number of cases. After the imputation, we excluded 7 participants with missing values for medical insurance and 3 on smoking from the descriptive sample, leading to a final sample of 10,739 respondents for the regression analysis of the factors influencing multimorbidity patterns.

### Statistical analysis

LCA was performed to identify the clustering pattern of different chronic diseases among the 10,749 participants based on 14 conditions. LCA is a useful tool for determining groups or subtypes of cases in multivariate categorical data. LCA assigns individuals into distinct and mutually exclusive multimorbidity classes based on the model-based posterior membership probabilities in our setting [28]. We examined two to six classes, and the best fitting solution was selected based on our evaluation of a variety of model fit statistics.

After selecting the best fitting solution and individuals' classifications into different classes, multinomial logit analysis was used to examine the influencing factors of multimorbidity classes, with all selected sociodemographic and lifestyle characteristics entered into the model simultaneously. All analyses were performed using Mplus version 6.1 and Stata version 17.

## Results

### Sample characteristics

Table 1 reports the characteristics of 10,749 participants used for LCA. The mean age was 69.1, with 31.9% aged 60–64, 28.3% aged 65–69, and 39.8% aged 70 and above. There was a slightly higher proportion of women (51.2 vs. 48.8). Among all participants, 6,489 (60.4%) lived in a rural area, and more than half (54.3%) had an education level less than primary school. There was a high coverage rate (96.9%) of medical insurance; however, more than 60% were covered by NRCMS with a limited benefit package and low reimbursement rate.

**Table 1:** Sample characteristics (*n* = 10,749)

Characteristics		Mean(SD)/N(%)
Age, mean (SD)		69.1 (7.1)
Age category, N (%)	60–64	3431 (31.9)
	65–69	3045 (28.3)
	70 +	4273 (39.8)
Sex, N (%)	Male	5241 (48.8)
	Female	5508 (51.2)
Marital status, N (%)	Unmarried	2396 (22.3)
	Married	8353 (77.7)
Residence, N (%)	Rural	6489 (60.4)
	Urban	4260 (39.6)
Education, N (%)	Less than primary	5832 (54.3)
	Primary and above	4917 (45.7)
Medical insurance, N (%)	No MI	334 (3.1)
	UEBMI	1590 (14.8)
	RMI	1745 (16.2)
	NRCMS	6799 (63.3)
	Other MI	274 (2.6)
Income Quintiles, N (%)	1	1936 (18.0)
	2	1946 (18.1)
	3	1915 (17.8)
	4	1932 (18.0)
	5	1932 (18.0)
	Missing	1088 (10.1)
Smoking status, N (%)	Never	6010 (55.9)
	Past or current	4734 (44.1)
Alcohol consumption, N (%)	Never	5665 (52.7)
	Past or current	5079 (47.3)
Physical activity, N (%)	None	1409 (13.1)
	Light	3691 (34.3)
	Moderate	2924 (27.2)
	Intensive	2722 (25.3)
Mean number of diseases, mean (SD)		2.6 (2.0)
Number of diseases, N (%)	0	1449 (13.5)
	1	2251 (20.9)
	2	2270 (21.1)
	3	1802 (16.8)
	4	1243 (11.6)
	5	801 (7.5)
	6	463 (4.3)
	≥ 7	470 (4.4)
Multimorbidity, N (%)	No	3700 (34.4)
	Yes	7049 (65.6)

*MI* is short for medical insurance. UEBMI refers to urban employee basic medical insurance (UEBMI) for urban employees and retirees, *NRCMS *refers to the new rural cooperative medical scheme (NRCMS) for the rural population, *RMI* includes the urban resident basic medical insurance (URBMI) for the urban unemployed people, and urban rural resident medical insurance (URRMI) in regions where integrated the NRCMS and URBMI

### Prevalence of multimorbidity and multimorbidity patterns

Table 1 also reports the prevalence of multimorbidity. The results indicated a high prevalence of multimorbidity in China, with 7,049 (65.6%) participants identified as having multimorbidity (at least two chronic conditions). Moreover, only 1,449 (13.5%) participants were free of any of 14 selected chronic diseases, whereas more than a quarter (27.8%) had four or more chronic diseases.

Table 2 lists the prevalence of different chronic diseases and the proportion of multimorbidity for each of the 14 chronic diseases included in the analysis. Hypertension (47.2%), arthritis (44.7%), and stomach disease (31.6) were the most prevalent diseases. Among the 7,049 multimorbid patients, hypertension was the most prevalent coexisting condition (in over 60% of multimorbid patients), followed by arthritis (58.8%), stomach disease (43.5%), chronic heart disease (36.6%), and dyslipidemia (35.8%).

**Table 2:** The prevalence and characteristics of certain chronic disease

Diseases	Prevalence *N* = 10,749		Participants with multimorbidity *N* = 7049	
	%	95% CI	%	95% CI
Hypertension	47.2	[46.3, 48.1]	63.0	[61.9, 64.1]
Dyslipidemia	24.5	[23.7, 25.4]	35.8	[34.7, 36.9]
Diabetes	15.4	[14.7, 16.0]	22.3	[21.4, 23.3]
Cancer	2.6	[2.3, 2.9]	3.6	[3.1, 4.0]
Chronic lung disease	19.0	[18.3, 19.8]	27.2	[26.2, 28.2]
Chronic liver disease	7.3	[6.8, 7.8]	10.6	[9.9, 11.3]
Chronic heart diseases	25.0	[24.2, 25.8]	36.6	[35.5, 37.7]
Stroke	10.2	[9.6, 10.8]	14.9	[14.1, 15.7]
Kidney disease	11.6	[11.0, 12.2]	17.0	[16.1, 17.9]
Stomach disease	31.6	[30.7, 32.5]	43.5	[42.3, 44.6]
Emotional problems	3.8	[3.4, 4.1]	5.4	[4.9, 5.9]
Memory-related diseases	6.1	[5.7, 6.6]	9.0	[8.3, 9.7]
Arthritis	44.7	[43.7, 45.6]	58.8	[57.7, 60.0]
Asthma	8.0	[7.5, 8.5]	12.0	[11.2, 12.8]

**Fig. 1:**
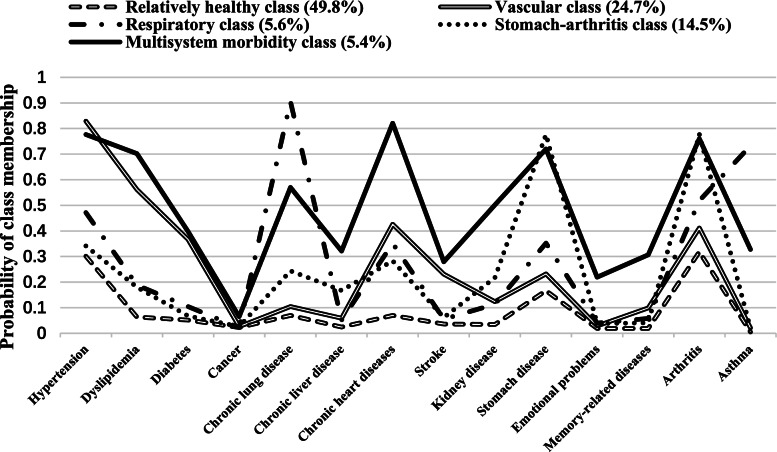
Five-class model of multimorbidity patterns

When identifying the clustering of diseases, two- to six-class models were examined using LCA. The five-class model emerged as the best fitting one (See Supplementary Table 1 for the model-fit statistics) and had the most reasonable clinical results for interpretability. Overall, all 10,749 participants were classified into one of the five classes. Based on the excess item response probability compared to the population average (Fig. 1 and Supplementary Table 2), we named the five classes: relatively healthy class, vascular class, respiratory class, stomach-arthritis class, and multisystem morbidity class. The relatively healthy class included participants with a substantially lower prevalence of all chronic conditions. The multisystem morbidity class consists of individuals with a substantially higher prevalence of all conditions. The vascular class included participants with a higher prevalence of hypertension, dyslipidemia, diabetes, chronic heart disease, and stroke. The respiratory class included individuals with a high prevalence of chronic lung diseases and asthma. The stomach-arthritic class was composed of individuals with a higher prevalence of arthritis and stomach or other digestive diseases. As shown in Fig. 1, nearly half (49.8%) of the participants belonged to the relatively healthy class, while 5.4% of participants were in the multisystem morbidity class. About 24.7%, 14.5%, and 5.6% of the participants were assigned to the vascular class, stomach-arthritis class, and respiratory class, respectively.

### Associated factors of multimorbidity patterns

Compared with participants belonging to the relatively healthy class, those classified into the multisystem morbidity class were older, most likely to be female and unmarried, and had lower education (see Supplementary Table 3). Table 3 presents the results of the associated factors of multimorbidity patterns, reporting the relative risk ratio (RRR) and 95% CI. Compared with those in the 60–64 age group, participants in the 65–69 age group and 70 + age group were more likely to be classified into multisystem morbidity class (RRR = 1.37 and 1.46, respectively), vascular class (RRR = 1.33 and 1.36, respectively), and respiratory class (RRR = 1.54 and 2.17, respectively). Women had a significantly higher probability of being in almost all multimorbidity classes (compared with the relatively healthy class), except for the respiratory class, with RRRs of 1.56 (95% CI: 1.35–1.81), 1.65 (95% CI: 1.38–1.98), and 1.90 (95% CI: 1.44–2.50) for the vascular class, stomach-arthritis class, and multisystem morbidity class, respectively.

**Table 3:** Multinomial logistic analysis of factors associated with the latent class

Latent class									
	Relatively healthy class	Vascular class		Respiratory class		Stomach-arthritis class		Multisystem morbidity class	
Independent variables	RRR	RRR	95% CI	RRR	95% CI	RRR	95% CI	RRR	95% CI
**Age category (ref = 60–64)**									
65–69	1.00	1.33^***^	[1.17,1.51]	1.54^***^	[1.21,1.97]	1.13	[0.98,1.31]	1.37^**^	[1.08,1.74]
70 +	1.00	1.36^***^	[1.21,1.54]	2.17^***^	[1.73,2.72]	1.17^*^	[1.01,1.35]	1.46^**^	[1.17,1.84]
**Sex (ref = male)**									
Female	1.00	1.56^***^	[1.35,1.81]	1.25	[0.96,1.64]	1.65^***^	[1.38,1.98]	1.90^***^	[1.44,2.50]
**Marital status (ref = unmarried)**									
Married	1.00	1.07	[0.95,1.21]	1.00	[0.81,1.24]	1.03	[0.89,1.19]	0.87	[0.71,1.08]
**Residence (ref = rural)**									
Urban	1.00	1.25^***^	[1.12,1.40]	0.81^*^	[0.67,1.00]	0.90	[0.78,1.02]	0.92	[0.75,1.13]
**Education (ref = less than primary)**									
Primary and above	1.00	1.20^**^	[1.08,1.34]	1.09	[0.90,1.31]	0.95	[0.83,1.08]	1.04	[0.85,1.27]
**Medical insurance (ref = no MI)**									
UEBMI	1.00	2.49^***^	[1.76,3.53]	1.32	[0.74,2.37]	1.09	[0.74,1.59]	2.03^*^	[1.16,3.54]
RMI	1.00	1.65^**^	[1.19,2.30]	1.35	[0.80,2.28]	0.93	[0.66,1.31]	1.40	[0.84,2.32]
NRCMS	1.00	1.54^**^	[1.12,2.10]	1.25	[0.76,2.04]	1.04	[0.76,1.42]	1.03	[0.64,1.66]
Other MI	1.00	2.45^***^	[1.60,3.76]	1.22	[0.56,2.69]	1.49	[0.92,2.42]	2.21^*^	[1.11,4.40]
**Income Quintiles (ref = 1)**									
2	1.00	1.24^**^	[1.05,1.46]	1.23	[0.93,1.63]	1.00	[0.83,1.20]	0.85	[0.64,1.13]
3	1.00	1.22^*^	[1.03,1.44]	1.46^**^	[1.10,1.93]	1.11	[0.92,1.33]	0.90	[0.68,1.20]
4	1.00	1.15	[0.97,1.36]	1.16	[0.86,1.57]	0.99	[0.81,1.19]	0.82	[0.61,1.10]
5	1.00	1.25^*^	[1.03,1.52]	0.98	[0.68,1.42]	0.86	[0.67,1.09]	0.73	[0.51,1.03]
Missing	1.00	0.95	[0.78,1.16]	1.00	[0.71,1.41]	1.04	[0.84,1.29]	0.79	[0.57,1.11]
**Smoking status (ref = never)**									
Past or current smoker	1.00	0.95	[0.83,1.09]	2.07^***^	[1.62,2.65]	1.05	[0.89,1.24]	1.48^**^	[1.15,1.90]
**Alcohol consumption (ref = never)**									
Past or current drinker	1.00	1.03	[0.92,1.15]	0.94	[0.78,1.14]	1.12	[0.98,1.28]	0.99	[0.81,1.22]
**Physical activity (ref = none)**									
Light	1.00	0.87	[0.75,1.02]	0.85	[0.66,1.09]	0.99	[0.82,1.21]	0.69^**^	[0.54,0.88]
Moderate	1.00	0.76^**^	[0.65,0.90]	0.74^*^	[0.56,0.97]	1.02	[0.83,1.25]	0.53^***^	[0.41,0.70]
Intensive	1.00	0.51^***^	[0.43,0.60]	0.49^***^	[0.36,0.65]	1.10	[0.90,1.34]	0.40^***^	[0.30,0.53]

*MI* is short for medical insurance. *UEBMI* refers to urban employee basic medical insurance (UEBMI) for urban employees and retirees, *NRCMS* refers to the new rural cooperative medical scheme (NRCMS) for the rural population, *RMI* includes the urban resident basic medical insurance (URBMI) for the urban unemployed people, and urban rural resident medical insurance (URRMI) in regions where integrated the NRCMS and URBMI. ^*^ *p* < 0.05, ^**^ *p* < 0.01, ^***^ *p* < 0.001

SES factors like urban residence and higher education were positively associated with the vascular class. Those living in urban areas were 1.25 times (95% CI: 1.21–1.40) more likely to be classified into the vascular class (compared with the relatively healthy class). Individuals with higher education (primary and above) were associated with 1.20 times (95% CI: 1.08–1.34) higher probability being in the vascular class. The relationship was further evidenced by the significantly higher odds of belonging to the vascular class for those with higher income levels (RRR = 1.25 for the highest quintile, 95% CI: 1.03–1.52). Compared to having no insurance, all types of insurance were associated with an increased likelihood of being in the vascular class, with the highest RRRs for UEBMI (RRR = 2.49, 95% CI: 1.76–3.53). In addition, the probability of being classified into the multisystem morbidity class was 2.03 times (95% CI: 1.16–3.54) higher for those with URBMI.

Lifestyle factors were also found to be important influencing factors. The probability of being assigned to the respiratory class increased by 2.07 times (95% CI: 1.62–2.65) for smokers compared with those who had never smoked. Also, smokers were more likely to be in the multisystem class (RRR = 1.48, 95% CI: 1.15–1.90). Physical activity decreased the probability of belonging to almost all other multimorbidity classes (compared with the relatively healthy class), except for the stomach-arthritis class. The results showed that intensive physical activity was associated with lower odds of being assigned to the vascular class, respiratory class, and multisystem morbidity class, with RRRs of 0.51 (95% CI: 0.43–0.60), 0.49 (95% CI: 0.36–0.65), and 0.40% (95% CI: 0.30–0.53), respectively. However, there were no significant influences of alcohol consumption on multimorbidity patterns.

## Discussion

Using the latest wave of CHARLS, this study reported the prevalence of multimorbidity patterns and examined its associated factors among elderly population in China. About 65.6% of all participants had at least two chronic conditions. By applying LCA, all participants were assigned to five multimorbidity classes based on their shared disease pattern: relatively healthy, vascular, respiratory, stomach-arthritis, and multisystem morbidity. When examining the associated factors of multimorbidity patterns, we found that participants of older age and female sex were more likely to be assigned to the vascular class and multisystem morbidity class (with the relatively healthy class as reference). A positive association was shown between SES and the vascular class. Also, the probability of being assigned to the multisystem morbidity class was significantly higher for those with UEMI or other MI. Smokers were more likely to be in the respiratory class, and multisystem morbidity class. Physical activity was found to decrease the probability of being in the vascular class, respiratory class, and multimorbidity patterns.

Multimorbidity was highly prevalent (65.6%) among the studied population, which slightly differed from the prevalence reported in the literature [13, 27, 29], largely due to different age restrictions and the inclusion of chronic conditions. For instance, Chen and colleagues [16] reported a rate of 45.5% among urban residents aged 45 and above using data from the 2011 wave of CHARLS, and Zhao and colleagues [27] reported a rate of 61.9% among individuals aged 50 and older in the 2015 wave of CHARLS. Our study provided reasonable estimates of the multimorbidity rate given the increased multimorbidity with age.

Using LCA, our study further identified five multimorbidity patterns among older adults in China, namely, relatively healthy (49.8%), vascular (24.7%), respiratory (5.6%), stomach-arthritis (14.5%), and multisystem morbidity (5.4%). Previous studies also studied multimorbidity patterns in China but more focused on disease similarities instead of categorizing all participants into exclusive multimorbidity classes [30, 31]. They found similar disease patterns to the present analysis, such as cardiovascular/metabolic clusters [31]; however, they seldomly identified the relative healthy class or multimorbidity class due to different focuses and cluster methods. Almost half of the participants were classified into the relatively healthy class in the present study. This coincided with previous studies using the same method showing a large proportion of participants in the relatively healthy class, with 63.8% in Span [12], 60.4% in Korea [32], 71% in England [26], and 24.7% in the United States [2]. In our analysis, 24.7% were classified into the vascular class consisting of participants with an excess rate of cardiovascular diseases. Similarly, studies in developed countries identified a metabolic or cardiovascular class [2, 12, 26, 32]. The existence of a respiratory class (5.6%) characterized by the high prevalence of chronic lung disease and asthma was evident, given the shared etiology [33]. Similar to previous studies revealing a digestive-kidney-arthritis cluster in China [15], our study also identified the stomach-arthritis class, which had not been reported in previous studies in other regions. One possible reason might be that some of the existing studies did not include stomach or digestive disease in the analysis. Other explanations might be that both arthritic and digestive diseases have a high prevalence in China [14] and the medication for arthritics (i.e., non-steroidal anti-inflammatory drugs) may lead to adverse side effects for the stomach [34]. The multisystem morbidity class was revealed in most studies, indicating that chronic diseases with diverse etiologies may also be clustered together [4].

Based on previous studies on the risk factors of multimorbidity, we extended the literature for multimorbidity patterns. Consistent with previous evidence [2, 12, 26, 32], participants with an older age had higher odds of multimorbidity for all classes, which is not surprising given the deterioration of health during the natural aging process. Regarding sex differences, there were inconsistent findings regarding multimorbidity prevalence [16, 17, 35], largely due to their inclusion of different chronic conditions and covariates. The sex difference could be better understood by looking at specific multimorbidity patterns. Consistent with previous studies [2, 12, 32], we found a higher probability of belonging to the vascular class, stomach-arthritis class, and multisystem morbidity class (compared to the relatively healthy class) for women. Similarly, previous studies also demonstrated that the prevalence of cardiovascular diseases [36], arthritis [37], and multimorbidity [27] was higher among women.

In contrast to previous studies in developed countries showing that people with low SES were more likely to belong to multimorbidity classes related to cardiometabolic conditions [12, 26, 32], we documented a significantly higher probability of the vascular class for those with higher SES in China, including urban residence, higher education, higher income, and medical insurance. Similarly, previous studies in China found a higher prevalence of multimorbidity for urban residents than their rural counterparts [14, 35]. This could be explained by people in developing regions, including China, with high SES being able to afford the consumption of high-calorie foods and avoid physically demanding tasks [38, 39], which may lead to a high prevalence of chronic diseases such as diabetes, hypertension, and dyslipidemia among those in urban areas and with a high income. Another reason might be the limited access to healthcare resources for those in disadvantaged SES groups, and the underdiagnosis of chronic diseases [40, 41].

In line with the existing evidence [26], we revealed that smoking and a lack of physical activity were risk factors for multimorbidity. More specifically, ever smoking was significantly related to the respiratory class and high multisystem morbidity class, which is plausible given the detrimental effects of smoking on the respiratory system [42]. People who participated in physical activity reported lower odds of being assigned to the vascular class, respiratory class, and multisystem morbidity class. This could be explained by physical activity benefitting cardiorespiratory function [43]. We did not find any significant influences of alcohol consumption on multimorbidity patterns, given the possible health benefits of light alcohol intake [44].

The current study applied LCA to classify the participants into five latent classes and analyze the associated factors of multimorbidity patterns. Nevertheless, it has several limitations. First, most of the variables are self-reported, which may cause potential bias. For example, all 14 chronic conditions come from respondent self-reporting. Therefore, the multimorbidity prevalence was underestimated, and associated factors for multimorbidity patterns should be interpreted with caution regarding the problem of underdiagnosis. Second, the cross-sectional design of the current study made it impossible to draw confident causal conclusions. Third, other potentially influential factors such as the nutrition status were not involved due to the limitations of the secondary dataset. Our strengths include the provision of the recent picture of multimorbidity picture based on the latest data from a nationally representative survey in China, which could be generalized to the whole country, and more importantly, the examination of risk factors for different multimorbidity classes, which has great implications for both health policy and clinical practice.

## Conclusion

In conclusion, using nationally representative data of older adults in China, we revealed a multimorbidity rate of 65.6%. LCA analysis showed that different conditions clustered together in a predictable pattern and identified five multimorbidity classes: relatively healthy, vascular, respiratory, stomach-arthritis, and multisystem morbidity. Older age, female sex, high SES, smoking, and no physical activity were risk factors for certain multimorbidity patterns. The findings have significant policy and clinical implications concerning the choice of prevention interventions and treatment strategies to target a multimorbidity pattern instead of a single condition.

## Supplementary Information

**Additional file 1:** **Supplementary Figure 1.** Flow chart of sample selection. **Supplementary Table 1.** Characteristics of participants in the regression analyses by latent class membership A Comparison of the fit statistics of models of latent class analysis. **Supplementary Table 2.** Item response probability from the five-class model. **Supplementary Table 3.** Characteristics of participants in the regression analyses by latent class membership.  

## Data Availability

The data used in this study are released data by CHARLS for public use. Permissions were acquired to access the data used in our research, which were granted by CHARLS team. The raw data is available on website (http://charls.pku.edu.cn/en).
