# Supplementary figures and images for: Multimorbidity patterns and associated factors in older Chinese: results from the China health and retirement longitudinal study
Source: BMC Geriatr. 2022 Jun 1;22:470. doi: 10.1186/s12877-022-03154-9 (PMC9158229; doi:10.1186/s12877-022-03154-9)

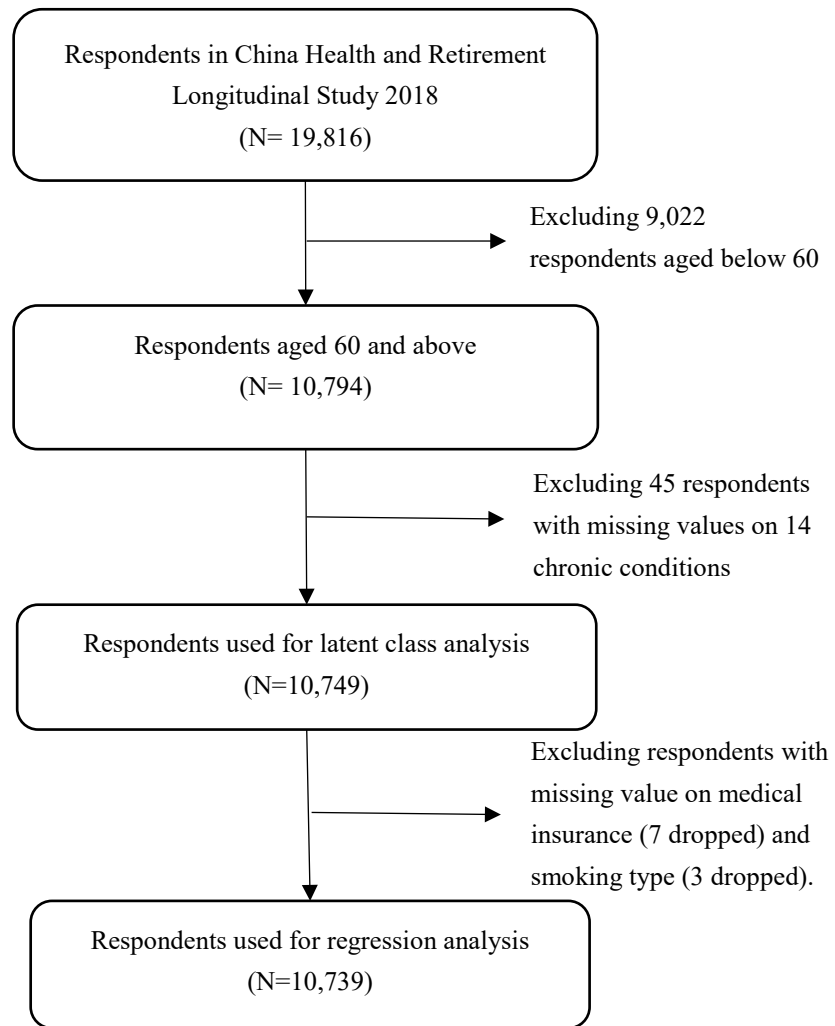

Supplement: Supplementary file 1 — Additional file 1: Supplementary Figure 1. Flow chart of sample selection. Supplementary Table 1. Characteristics of participants in the regression analyses by latent class membership A Comparison of the fit statistics of models of latent class analysis. Supplementary Table 2. Item response probability from the five-class model. Supplementary Table 3. Characteristics of participants in the regression analyses by latent class membership. [file 12877_2022_3154_MOESM1_ESM.pdf]
